# Dog eye movements are slower than human eye movements

**DOI:** 10.16910/jemr.12.8.4

**Published:** 2020-02-05

**Authors:** Soon Young Park, Catarina Espanca Bacelar, Kenneth Holmqvist

**Affiliations:** Comparative Cognition, Messerli Research Institute, University of Veterinary Medicine Vienna Medical University of Vienna, University of Vienna, Austria; Department of Psychology, Regensburg University, Germany; Department of Psychology, Nicolaus Copernicus University, Torun, Poland; Department of Computer Science, University of the Free State, Bloemfontein, South Africa; UPSET, NWU Vaal, South Africa; Faculty of Arts, Masaryk University, Brno, Czech Republic

**Keywords:** eye movement, dogs, eye-tracking, saccades, fixations

## Abstract

Eye movement of a species reflects the visual behavior strategy that it has adapted to during its evolution. What are eye movements of domestic dogs (Canis lupus familiaris) like? Investigations of dog eye movements per se have not been done, despite the increasing number of visuo-cognitive studies in dogs using eye-tracking systems. To fill this gap, we have recorded dog eye movements using a video-based eye-tracking system, and compared the dog data to that of humans. We found dog saccades follow the systematic relationships between saccade metrics previously shown in humans and other animal species. Yet, the details of the relationships, and the quantities of each metric of dog saccades and fixations differed from those of humans. Overall, dog saccades were slower and fixations were longer than those of humans. We hope our findings contribute to existing comparative analyses of eye movement across animal species, and also to improvement of algorithms used for classifying eye movement data of dogs.

## Introduction

Visuo-cognitive abilities of domestic dogs have been an important topic for many researchers (1). In these studies, researchers infer cognitive traits of dogs from the characteristics of their visual behavior. Video-based remote eye tracking systems which became readily available in the mid-1990s changed the way such studies were made (2). Formerly, studies of dog visual cognition depended on macro-scale visual behavior data, namely visual inspection of head and gaze direction of dogs by human coders (3, 4, 5, 6, 7, 8, 9, 10). When instead using video-based eye-tracking systems, researchers were able to acquire and use data of eye movements such as saccades and fixations at much finer temporal and spatial resolution than were possible to collect by human coding. When coupled with increased convenience in data acquisition and processing provided by such systems, more diverse and detailed aspects of dog visual cognition could be explored (11, 12, 13, 14, 15, 16, 17).

Surprisingly, however, investigations of dog eye movements themselves, the very measurements used in the eye-tracking studies, have been scarce. To our best knowledge, less than a handful of studies have investigated dog eye movements as such (18, 19, 20). Despite their importance, the data in these previous studies are limited to manually coded blinks of awake dogs or involuntary saccades observed in anaesthetized dogs. Although more dog eye movement recordings can be found in clinical studies, the data in such studies are of abnormal involuntary eye movements such as nystagmus seen in dogs with certain ophthalmic or neurological conditions (21, 22, 23). Hence, the fundamental characteristics of non-clinical voluntary dog eye movements themselves seem to have not been explored to date. Especially, studies of saccade and fixation characteristics in awake stimuli-viewing dogs are non-existent. This void contrasts with the vast amount of literature on eye movement characteristics of other animal species such as non-human primates, rodents, birds, reptiles, and felines that appear in reviews (24, 25, 26, 27).

Previous work on other species and its comparative analyses has revealed important information on eye movements, especially that of saccades. The comparative analyses have revealed that the function and fundamental characteristics of saccades are shared across diverse phyla, ranging from crustacean to humans, yet the details of eye movements differ across species (25, 26). The differences reflect that eye movements are species uniquely adapted to optimize the efficiency of their visual and other systems interacting with the visual information from their habitat (24, 25, 26). The shared saccadic characteristics among the species could be readily observed in primates, cats, goldfish, zebrafish, and rabbits (28, 29, 30, 31, 37). Their saccades exhibit systematic relationships among the saccadic metrics such as that of peak velocity and amplitude (the main sequence) and that of duration and amplitude (Carpenter’s relationship) (33, 34). Yet, the details of each relationship as well as the quantities of each saccadic metric such as amplitude, peak velocity, and duration appeared to be different among the species which echoes species-specific optimization of saccade strategy (Table 1 and Table 2).

**Table 1:** The linear regression slopes of the main sequence of diverse animal species. The slopes are either the reported results (averaged if multiple) or our approximations based on the figures (only considering the results of saccades ≤ 20 °) in the studies.

	Main sequence (deg/sec per deg)	
Species	Slope	Publications
human	≈ 15	Berg et al. (2009, Fig.3)
	≈ 20	Boghen et al. (1974, Fig.2)
monkey	≈ 33	Fuchs (1967, Fig.5 & Fig.16)
	≈ 33	Berg et al. (2009, Fig.3)
cat	≈ 13.6	Evinger & Fuchs (1978, Fig.3)
rabbit	≈ 13	Collewijn (1970, Fig.5)
goldfish	21	Easter Jr (1975, Fig.5)
zebrafish	≈ 11*.*2	Chen et al. (2016, Tab.1)

**Table 2:** The linear regression slopes of Carpenter’s relationship of diverse animal species. The slopes are either the reported results (averaged if multiple) or our approximations based on the figures (only considering the results of saccades ≤ 20 °) in the studies.

	Carpenter’s relationship (ms/deg)	
Species	Slope	Publications
human	≈ 2.2	Robinson (1964, Fig.3)
	2.2	Fuchs (1967, Fig.15)
monkey	1.1	Fuchs (1967, Fig.15)
cat	≈ 3*.*1	Evinger & Fuchs (1978, Fig.3)
goldfish	≈ 2.8	Easter Jr (1975, Fig.5)

The former studies have called the need for more interdisciplinary comparative analyses involving diverse animal species to explore saccade mechanisms and its evolution (24). The domestic dog is a species of Canidae that could add a great benefit to such analysis. Dogs share many aspects of eye movement system with primates. Also their habitat, behavioral repertoire, and evolutionary history are relatively well-known.

Their high trainability is convenient for researchers to bring out voluntary target-selecting saccades (in contrast to involuntary reflexive saccades) and fixations in dogs during natural-viewing visual tasks, which has not been easy to observe in many other non-primate animals.

Yet, there is an additional need for investigating dog eye movements, which concerns the usage of video-based eye-tracking systems. When using video-based eye-tracking systems, the included software automatically processes raw eye movement data, consisting of samples of x, y (screen gaze coordinates), and time in milliseconds, into ready-to-use eye movement data in the form of fixations and saccades. Researchers subsequently use the processed data to test their hypotheses and draw results and conclusions. It is important to note that each eye-tracking system uses its own eye movement detection algorithm and related threshold settings to categorize the raw data into fixations and saccades. Thus, the performance of each algorithm affects how close the eye-tracking study results are to reality. Currently, the default algorithm and threshold settings provided by manufacturers are exclusively developed for tracking the movements of adult human eyes. If dogs move their eyes differently from humans or their eye movement data characteristics differ from those of human adults, the performance of the algorithm and its threshold settings is likely not optimal. The suboptimal performance of such standard algorithms and threshold settings have been demonstrated in the studies with non-adult human subjects (39, 40, 41, 42). Therefore, information on dog eye movement and their data characteristics is required, especially that based on the data collected in free viewing tasks, to evaluate and improve current default algorithms and threshold settings used in dog eye tracking.

As a means to answer the needs mentioned above, we have collected eye movement data of dogs using a video-based eye-tracking system. Human data were also collected for comparative analyses between the two species, with the same experimental design, apparatus, and stimuli. The data of both species were processed with a custom-made velocity-based event detection algorithm to detect their saccades and fixations. We first investigated whether dog saccades share the common systematic relationships among saccadic metrics observed in humans and other animal species mentioned above. Then, we compared how similar or different the parameters of the relationships and each saccade metric are between humans and dogs. Finally, we discussed dog eye movement characteristics in relation to their morphology and evolutionary history compared to those of humans and other animals.

## Methods

All experimental procedures were approved in accordance with GPS guidelines and national legislation by the Ethical Committee for the use of animals in experiments at the University of Veterinary Medicine Vienna (Ref: 09/08/97/2012) and by the Ethical Committee of Medical University of Vienna for human experiments (No. 1336/2013).

### Participants

We recruited dog participants by contacting dog owners who previously shared their contact information for possible participation. Initially, 33 dogs were invited for pre-experiment training. Eight dogs were excluded because of their eyelid conditions (pronounced third eyelids or droopy eyelids) that could result in poor data quality. Further, five dogs could not continue the pre-experiment training which left us 20 dog participants available for the experiment (age: m = 5.2 years, SD = 2.6 years; gender: eight males, twelve females). Yet, due to their limited availability for two visits, only nine dogs could complete all experiment trials. The dog participants were one Akita Inu, one Australian Shepherd, five Border Collies, one Boxer, one Petit Brabancon, one Golden Retriever, two Siberian Huskies, one Jack Russell Terrier, one Parson Russell Terrier, two Rhodesian Ridgebacks, and four mixed breed ones. There were 16 human participants (age: m = 29.9 years, SD = 10.4 years; gender: seven males, nine females) of volunteering graduate students, dog owners or university staff with normal or corrected vision (glasses were off during the experiment) who completed all trials.

### Stimuli

Stimuli were either black and white figure (16) or color photo (24) images (human face, dog face, or round non-face objects), that were generated using the freely available image processing tool GIMP or collected from Radboud FACE Database and internet websites with their permission (43). The sizes of the images ranged from 246−411 (pixels) in width and 257−341 (pixels) in height which corre-sponded to the viewing angle of approximately 8−12 ° and 7−10 °, respectively. Each image was presented on the left or right side, 258 pixels horizontally off center of the screen. Unique random sequences of stimulus presentation order were created for each participant. The stimulus presentation was controlled by the experiment software generated by the visual experiment creation tool Experiment Builder of SR Research.

### Training of dogs 

**Figure 1.**
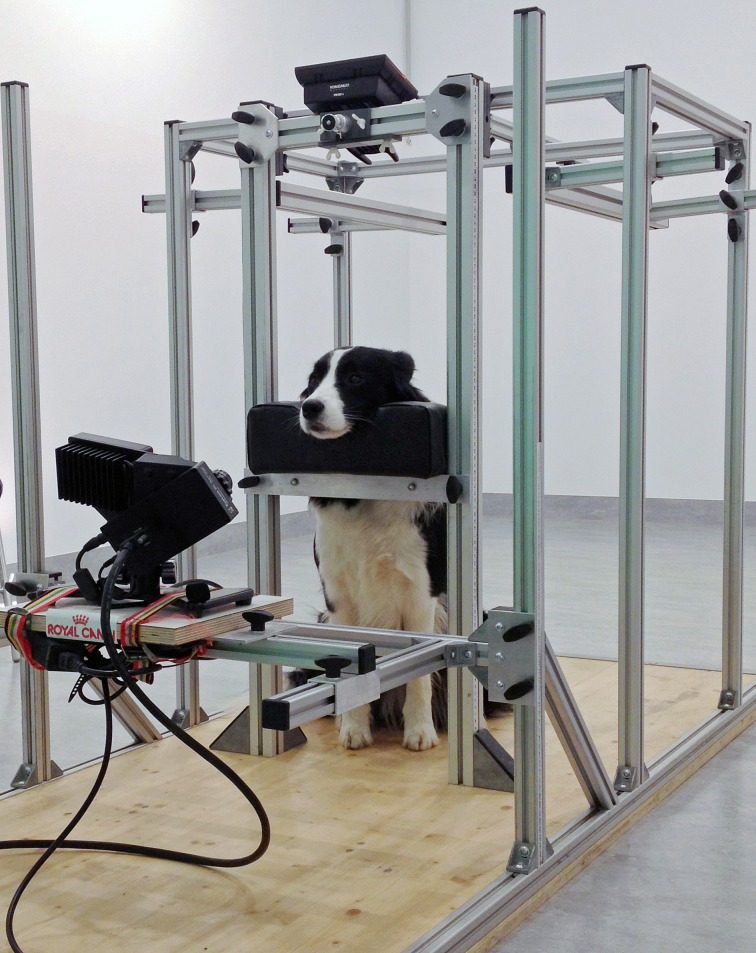
A dog participant in the chin rest.

In preparation of data recording, dog participants were trained by a professional dog trainer and the experimenter. The details of the training method depended on individual characteristics and owner preferences, but in principle we used a shaping method based on operant conditioning using positive reinforcement (a food reward) (44). The target behavior of the training was twofold: 1) keeping their chin on the chinrest, while 2) looking at a series of seven white light points, which appeared for approximately one second each on the screen (this is used in the calibration procedure), and continuously watching a video clip of animals or humans appearing on the screen for approximately 20 seconds. We reached the final target behavior through the following four steps by rewarding the dogs when their behavior gets close to the target behavior of each step in response to a command. The steps are:

1. The dog approaches the chin rest.

2. The dog puts its chin on the chin rest (Figure 1).

3. The dog keeps its chin on the chin rest for a required amount of time.

4. The dog watches the white light points and various video stimuli keeping its chin on the chin rest for a required amount of time.

At the same time, we trained the dogs exclusively for the calibration procedure. The calibration procedure requires the participant to look at the calibration points steadily and, at the same time, the experimenter to register eye images to the system (by pressing a key), when the participant appear to look at one of the points. Here, correct judgement of the timing is crucial. Wrong judgement of the timing produces poor calibration results and consequently introduces offsets in estimated gaze location results. Due to the requirements for both the participant and experimenter, the calibration results are known to be affected by experimenter experience and participant ability to follow the rather stringent instruction “look as precisely as you can at the points that you will see on the screen, and do not move the eye until the point disappears.” (45). Because of many reasons, the participants that appeared to look at the calibration points could actually shortly look away from the points without moving their head very close to the moment when the experimenter presses the key (2). Verbal confirmation of human participants that they are fixating on the calibration points helps the experimenters. Further, it has been demonstrated that the best quality calibration result is obtained when the participants themselves press the key (45).

 As expected, we were challenged with the impossibility of verbal communication between humans and dogs. The calibration training, namely light feedback training, was devised in an attempt to minimize the lack of verbal communication. In the training, dogs were trained to give us their behavior feedback of touching the calibration point location with their nose after looking at it in response to the trainer/experimenter’s cue. For this purpose, we crafted a screen device and a light torch attached with a clicker. The screen device, that mimicked the screen used during the actual experiment, consisted of three foldable wooden screens (2 m x 2 m each), resulting in three screen walls standing in front of a dog in a room. Using the same shaping training technique, the dogs were trained to press the calibration point (a light shone through the screen on a location by the experimenter behind the screen) with their nose to make clicker sound. This feedback training started with one light point and extended to a series of seven consecutive light points. The light point appeared randomly on one of the three screen walls to generalize the effect of the behavioral conditioning. Once the dogs consistently show their feedback behavior, we took it as the confirmation that the dogs do look at the points. At the end of the training, we discouraged them from touching the point locations, in preparation for the real calibration procedure in the experiment. The same light was used during the experiment, yet the dogs were not allowed to touch the light locations, and they continued to look at the screen, where a fixation point in the center appeared after the calibration procedure and before an experimental stimulus.

### Apparatus

The same apparatus was used for both human and dog participants. The room used for the eye movement recording was 3.6 m (w) x 6.0 m (l) x 3.0 m (h), windowless, quiet, and dimly lit (approx. 80 lx) using white LED lamps. Stimuli were back-projected on a 1.1 m x 0.8 m screen by a NEC M350XS projector (resolution: 1024 x 768 pixel array). Each participant sat or stood and placed their head in a custom metal frame structure that hosted a chinrest suitable for both dogs and humans. Once positioned onto the chinrest, viewing distance from the participants to the display area was 2 m. Practically, there was no physical restraint of a head on the chinrest, and we depended on the instruction or training given to humans or dogs, respectively. The experimenter controlled the experiment software and monitored the subjects from a control area behind the screen.

We used an EyeLink 1000 video-based eye tracker (SR Research) to record the movements of the right eye of the humans and dogs at 1000 Hz from a distance of 50-55 cm to the eye. According to the manufacturer the maximum head movement the eye tracker can allow without accuracy reduction is 2.5 cm horizontally and vertically, and 1 cm back and forth. Like all video-based eye-tracking systems, the working mechanism of EyeLink 1000 is based on the eye images with identified pupil and infrared light reflection on the cornea. As such, whether the system can track the eyes or not depends on how visible the pupil is and how correctly the corneal reflection is identified. The EyeLink 1000 takes the center of the pupil (P) in combination with the center of the corneal reflection (CR), both in camera pixel coordinates, and associates the calculated values of P−CR to the positions on the stimulus screen via a calibration. This robust working mechanism makes it possible for us to use EyeLink1000 to track dog eyes that share similar cornea and pupil morphology to humans’, despite that the system was initially developed for tracking human eyes. Yet, through monitoring camera view and the quality of raw data we could identify tracking interference in some dogs, that similarly occurs in human eye-tracking. Importantly, we also considered the possibility that the performance of default EyeLink 1000 eye movement event classification algorithm might not be optimal as it requires predetermining thresholds of certain saccade characteristics such as saccade velocity and acceleration, that are based on human saccade characteristics, for eye movement event classification. For this reason, we have used a custom-made algorithm to classify eye movement events in the raw data (see Data reduction section for more details).

### Experimental procedure

The pre-experimental training replaced verbal instruction for our dog participants. To minimize differences in pre-experimental conditioning between the two species, we instructed human participants only to refrain from head movements and look at the screen wherever they would like, except for the calibration points that they must look at. At the start of each experiment, a three-point calibration was performed to set up the eye tracker (for further details about the procedure, see Holmqvist et al., 2011(2)). At the start of each experiment, calibration results were validated and repeated until an average error less than 1.5 ° was achieved. Stimuli were presented in blocks with two trials in each block. Each trial started with a display containing a fixation point at the center of the screen. After the experimenter confirmed the eye location on the fixation point, a trial stimulus was presented for seven seconds, and the next trial started after another confirmation of the eye location on the fixation point. Therefore, a block consisted of a calibration procedure, fixation point confirmation, 1st stimulus for seven seconds, second fixation point confirmation, and 2nd stimulus for seven seconds which lasted for approximately 30 seconds. After each block, the dog or human participants could move or eat a food reward. To compensate the between-block head movement, we repeated the calibration procedure every block, but not the validation procedure. If there was obvious movement (head rotated more or out of the chin rest) within a block or a trial, we recalibrated the eye after the trial, unless it is during a stimulus presentation. Eye movement recording was stored to file for offline analysis. Given the average accuracy obtained during the training and the experiment (0.88 ° for dogs and 0.51 ° for humans), the average offset in the eye position data recorded by the eye tracker is expected to be around 28.2 pixels (3.1 cm) for dogs, and 16.3 pixels (1.81 cm) for humans. However, we should mention that for the purpose of the current study the accuracy of tracked areas in the stimuli was not vital to us.

**Table 3:** Number of saccades and fixations included in the analysis out of total number of data (analyzed data/total data).

	Species	
Eye movements	Human	Dog
Saccades	2667/8625	1475/2618
Fixations	7469/9164	1415/3095

### Data reduction

Processing of the recorded raw eye movement data (composed of time in ms, x and y screen coordinates) was performed offline with custom MATLAB (MathWorks) scripts of the Nyström & Holmqvist (2010) event classification algorithm, as implemented by Niehorster, Siu, & Li (2015) (46, 47). First, event detection was performed to classify the raw data into eye movement events such as blinks, saccades, post-saccadic oscillations and fixations. The algorithm detects saccades by means of a velocity threshold that is not predetermined, but adaptive to the local noise level in the eye movement data. That is, it requires users to set only an initial threshold that is motivated by physiological limitations of eye movements, and the initial threshold is automatically updated by the algorithm based on the level of noise within a trial, a participant or an experiment. By taking into account local noise level it can classify eye movement events more correctly than other velocity-based algorithms that do not, as it can avoid, for example, miss-classifying fixations with heavy noise as small saccades, when velocity threshold is set too small (46). All successfully recorded sequences of data longer than 50 ms that were not classified as saccades or blinks were classified as fixations. After the event detection, eye movement event data were further processed to be datasets of eye movement responses using Microsoft-Excel and R (version 3.5.1) (R Core Team, 2019).

### Data processing and statistical analysis

We excluded saccades with indications of erroneous measurements. Saccades were considered erroneous if their amplitude was more than 20 ° (the maximum visual angle of our stimulus display setting), if the peak velocity was more than 700 deg/s, or if the duration was more than 600 ms. Fixations not on the stimulus were also excluded. The amount of data included in the data analysis is shown in Table 3. For the saccadic skewness values, we used the measurement of Collewijn, Erkelens, & Steinman (1988): the acceleration phase (time to peak velocity) divided by the total saccadic duration (32). As Collewijn et al. (1988) used coil system, the difference in eye-tracking method between our and their studies might influence the values. All statistical models we used are of General or Generalized Linear, depending on the distribution types of the response variables, Mixed-Effects Model (LMM/GLMM) to account for the baseline differences in the repeated measurements of different individuals (48). The significance of the effects was judged by p-values with 0.05 threshold reported in type III analysis of variance (ANOVA) table (Wald chi-square tests) or pairwise comparisons results (two-tailed Z or Student’s t-tests) (49). The specifications of the statistical models are described in Table 4. All models are tested in R. The data and R scripts for statistical analysis are available at https://zenodo.org/deposit/3629654.

**Table 4:** Statistical models tested in the study.

Eye movement	Topic		Model specification (in Wilkinson notation)				family	Link function
Saccade	main sequence		peak velocity∼		amplitude ∗ species + (1 + amplitude | name)		Gaussian	identity
	Carpenter’s relationship		duration∼					
	velocity profile		time to peak velocity∼					
			deceleration duration∼					
	metrics		skewness∼		species + (1 | name)		Gamma	log
			amplitude∼					
			duration∼					
			peak velocity∼					
			velocity∼					
			skewness∼				Gaussian	identity
Fixation			duration∼				Gamma	log

## Results

### Saccades

**Figure 2.**
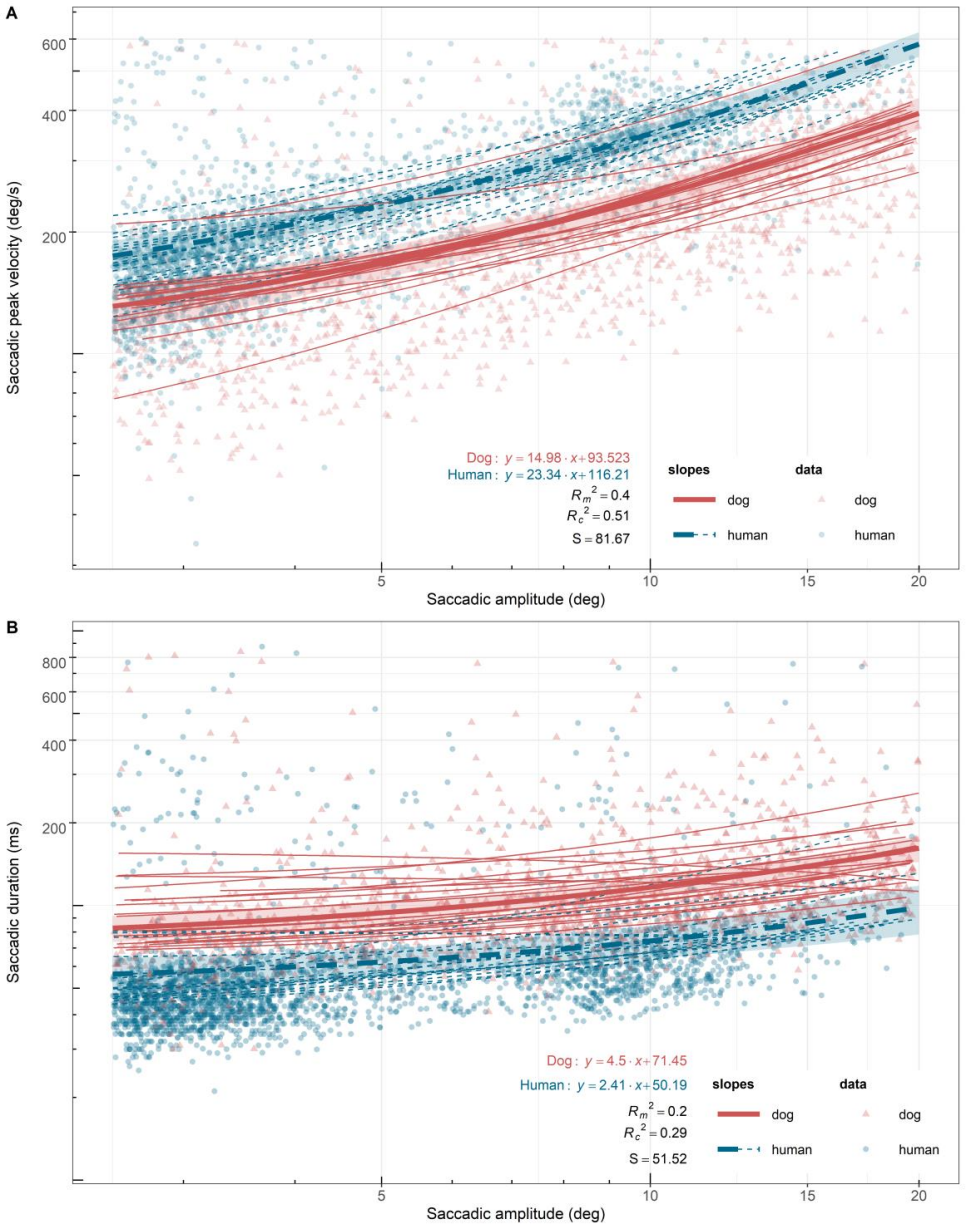
*Least square linear regression lines and equations of the main sequence (* *A* *) and Carpenter’s relationship (* *B* *) of human and dog saccades. The lines are plotted in log10−log10 scale for zooming purpose (without transformation of the original data), hence* *they appear curved. The thicker lines and the surrounding shadings visualize the lines of species means (marginal means) and 95% confidence intervals (+/ −1.96 ∗ SE) of the lines, respectively. Thinner lines visualize the lines of the individuals. Shapes indicate data used in the analysis. Goodness of fit statistics of the models: R*_*m*_^*2*^ *= marginal R* ^*2*^ *, R*_*c*_^*2*^ *= conditional R* ^*2*^ *, S = Standard deviation of the residuals.*

**Figure 3.**
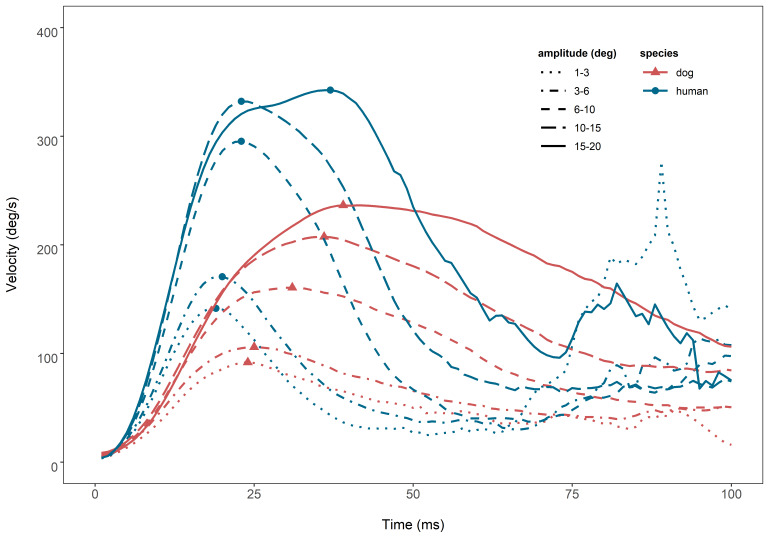
Comparison of saccadic velocity profiles of human and dog saccades in different amplitudes. The same patterns of velocity profiles as in the Figure 1 in Collewijn, Erkelens, & Steinman (1988) can be observed in both species saccades. The amount of acceleration duration (P<0.0001) and the duration of deceleration (P<0.002) increased for saccades that have bigger amplitudes in both species. However, human saccades more quickly accelerated to the peak velocity with increasing amplitude (P < 0.002) than dog saccades. Also, human saccades overall took less to decelerate than dog saccades (P < 0.002). Each profile line plots velocity means of each species saccades of a given amplitude. Solid symbols (triangles and points) indicate peak velocities of the profiles.

We first examined whether the saccades of dogs and humans show the systematic relationships between saccadic metrics previously reported in the studies of humans and other animal species. As expected, saccades of both species showed the typical pattern of the main sequence and Carpenter’s relationship, where the peak velocity (X^2^(1) = 211.14, P < 0.0001) and the duration (X^2^(1) = 71.60, P < 0.0001) of both species saccades significantly increased with amplitude, respectively. Yet, the slopes of the main sequence were significantly different between the two species. The main sequence (peak velocity-amplitude) slope of dog saccades was significantly smaller than that of human saccades (X^2^(1) = 30.23, P < 0.0001). The increase rate of peak velocity per 1 ° was 14.98 (SE = 1.52) for dog saccades and 23.34 (SE = 1.12) for human saccades (Figure 2A). However, the saccadic peak velocity offsets (intercepts) were not significantly different between the two species (dogs: 93.52 deg/s, SE = 11.60 deg/s; humans: 116.21 deg/s, SE = 8.01 deg/s). Furthermore, dog saccades did not show a clear inflection point, where the slope changes, while it appeared at around 10 ° in human saccades. There was a slightly bigger individual variation in the main sequence slopes among dogs than humans (dogs: SD = 3.58, humans: SD = 3.48), while in intercepts, the variation among human individuals was bigger (dogs: SD = 24.37, humans: SD = 25.80). The slopes of Carpenter’s relationship (duration-amplitude) between the two species also differed significantly. The slope of dog saccades was significantly larger than that of humans (X^2^(1) = 7.19, P < 0.008). The increase rate of duration per 1 ° was 4.50 (SE = 0.53) for dog saccades and 2.41 (SE = 0.78) for human saccades (Figure 2B). The saccadic duration offset of dog saccades was also significantly longer than that of humans (X^2^(1) = 11.88, P < 0.0006). On average, dog saccades took 21.27 ms (SE = 6.17) more for the same amplitude saccades (dogs: M = 71.45 ms, SE = 4.57 ms; humans: M = 50.19 ms, SE = 6.17 ms) (Figure 3). For both slopes and intercepts, individual variation among dogs was bigger (slopes: dogs: SD = 1.65, humans: SD = 1.48; intercepts: dogs: SD = 12.27, humans: SD = 10.63).

Further, we examined the skewness of saccadic velocity profiles, and the saccadic skewness values of the two species, in relation to the amplitudes of the saccades. The skewness of the saccadic velocity profile plots is known to increase as saccadic amplitude increases. We could observe such pattern in the saccades of both species (Figure 3). In the velocity profile plots of dogs and humans, the amount of acceleration duration, that is, time to peak velocity, and deceleration duration increased with their amplitudes. This made the skewness of the plots bigger, with right tails getting longer as amplitude increases (32). However, differences could also be observed in the details of the velocity profile plots. Human saccades more quickly and more powerfully accelerated to the peak velocity, and then dropped also quickly. Dog saccades never reached human peak velocity, but instead kept going for a longer time at a lower speed. The peak velocities of dog saccades were smaller than those of humans across all amplitudes. Also, dog saccades had longer acceleration duration to reach peak velocity than those of humans. In subsequent tests, we could confirm that both species saccades indeed took longer to reach peak velocity (X^2^(1) = 63.76, P < 0.0001) and to decelerate (X^2^(1) = 32.38, P < 0.0001) as amplitude increases. However, only the increase rate for time to peak velocity was significantly different between the two species, where it was higher for dog saccades than those of humans (X^2^(1) = 9.96, P < 0.002). The increase rate of time to peak velocity per 1 ° amplitude was 2.29 (SE: 0.29) for dog saccades and 0.96 (SE: 0.42) for human saccades. Similarly, the deceleration duration of dog saccades was overall significantly longer than that of human saccades (X^2^(1) = 10.68, P < 0.002). Conversely, the saccadic skewness values, calculated using time to peak velocity divided by total duration, are known to decrease as saccadic amplitude increases (32). In our data, the typical pattern was observed for human saccades, where the rate of decrease in saccadic skewness value per 1 ° was 0.158. Yet, dog saccades showed the opposite pattern, where their saccadic skewness value increased with the rate of 0.164. However, neither the slopes of both species, nor the difference between them were statistically significant.

**Figure 4.**
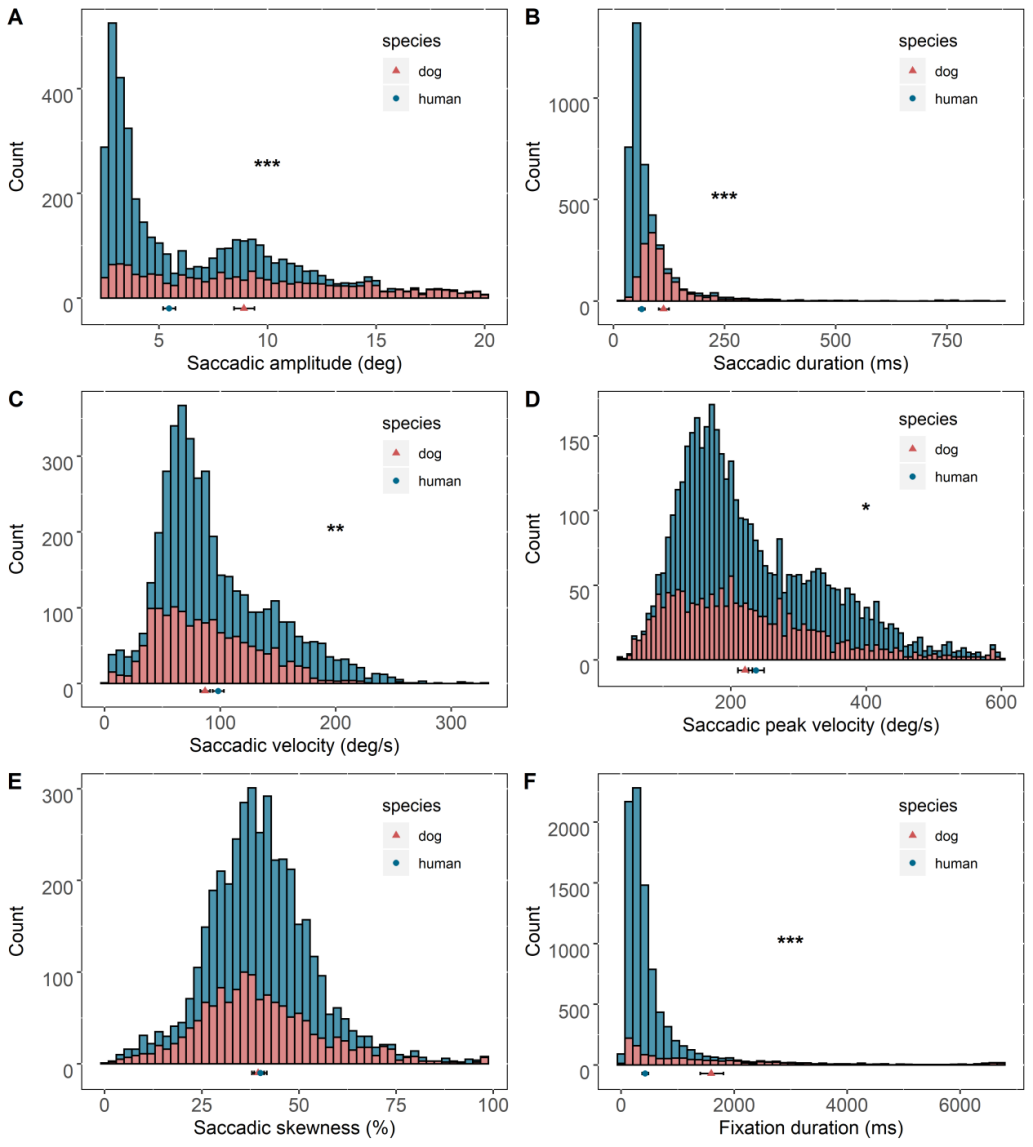
Comparison of saccadic and fixation metrics between humans and dogs. Histograms visualize distribution patterns of human and dog saccades or fixations. Solid shapes and error bars depict marginal means and 95% confidence intervals (+/−1.96∗SE), respectively. Significance codes: ‘***’ < 0.001 < ‘**’ < 0.01 < ‘*’ < 0.05.

Thereafter, we compared each saccade metric between the two species. Overall, the two species significantly differed in the amplitude, duration, peak velocity, and average velocity of their saccades. When seeing the same stimuli, dogs on average made bigger saccades than humans (Figure 4A). The average amplitude of dog saccades was 1.64 (95% CI = 1.53-1.77) times that of humans (Z = 13.37, P < .0001; dogs: M = 8.98 °, SE = 0.24 °; humans: M = 5.46 °, SE = 0.14 °). There was a bigger variation in the measures of saccadic amplitude in dogs than humans (dogs: SD = 0.80 °, humans: SD = 0.10 °). As similarly shown in Carpenter’s relationship, the average duration of dog saccades was also significantly longer, where the average of dogs was 1.74 (95% CI = 1.55-1.96) times that of humans (Z = 9.18, P < 0.0001; dogs: M = 109.75 ms, SE = 4.60 ms; humans: M = 63.01 ms, SE = 2.75 ms) (Figure 4B). There was a bigger variation in the measures of saccadic duration in dogs than humans (dogs: SD = 20.27 ms, humans: SD = 16.83 ms). On the other hand, as similarly shown in velocity profiles of both species, the average peak velocity of dog saccades was significantly smaller, 0.93 (95%CI = 0.86-1.00) times that of humans (Z = 2.09, P = 0.040; dogs: M = 223.67 deg/s, SE = 5.70 deg/s; humans: M = 241.30 deg/s, SE = 6.23 deg/s) (Figure 4C). Likewise, average velocity of dog saccades was also significantly smaller, 0.89 (95%CI = 0.83-0.98) times that of humans (Z = 3.17, P = 0.002; dogs: M = 87.3 deg/s, SE = 2.23 deg/s; humans: M = 97.9 deg/s, SE = 2.51 deg/s) (Figure 4D). There were bigger variations in the measures of saccadic peak velocity and average saccadic velocity in humans than dogs [peak velocity: dogs (SD = 24.75 deg/s), humans (SD = 28.04 deg/s); velocity: dogs (SD = 7.93 deg/s); humans (SD = 11.99 deg/s)]. On the other hand, the saccadic skewness values did not significantly differ between the two species, where that of dogs was slightly smaller (dogs: M = 39.53 %, SE = 0.76 %; humans: M = 40.02 %, SE = 0.78 %) (Figure 4E). There was a bigger variation in the measure of saccadic skewness in dogs than humans (dogs: SD = 2.93 %, humans: SD = 2.20 %).

### Fixations

The average duration of dog fixations was significantly longer than that of humans (Figure 4F), where the average duration of dog fixations was 3.76 (95%CI = 3.13 − 4.5) times that of humans (Z = 14.31, P < 0.0001; dogs: M = 1593.00 ms, SE = 104.55 ms; humans: M = 424.23 ms, SE = 27.64 ms). The variation in the measure was bigger in dogs than humans (dogs: SD = 399.0 ms, humans: SD = 313.6 ms).

## Discussion

### Shared, yet different eye movement characteristics between dogs and humans

With our data, two interesting observations of dog eye movements have been made. First, dog saccades follow the systematic relationships between saccade metrics previously shown in humans and other animal species. Typical patterns of the main sequence and Carpenter’s relationship could be observed in dog saccades. Second, the details of the main sequence, Carpenter’s relationship, velocity profiles, and the quantities of most of dog saccade metrics turned out to be different from those of humans. Overall, dog saccades were slower indicated by their lower peak velocity and average velocity, longer duration to reach peak velocity, and longer deceleration duration. Their fixations, on the other hand, were longer than those of humans.

The main sequence and Carpenter’s relationship slopes of dogs (14.98 deg/s per deg, 4.5 ms/deg) and humans (23.34 deg/s per deg, 2.41 ms/deg) we reported can be compared to those previous studies reported (Table 1 and Table 2). Both slopes of dogs seem arguably the closest to the averaged values of the two cats Evinger and Fuchs (1978) reported (29). On the other hand, both slopes of humans in our study were overall higher than those Boghen, Troost, Daroff, Dell’Osso, & Birkett (1974) and Berg, Boehnke, Marino, Munoz, & Itti (2009) reported (35, 36). Traditionally, the slope of the main sequence has been known to vary across studies due to diverse reasons such as differences in sample sizes, visual tasks, and notably eye movement recording systems used. For example, infrared pupil and corneal reflection based eye-tracking systems are known to give higher values than Electrooculography (EOG) (35). The difference in the recording systems might explain the marginally lower slopes Boghen et al. (1974) reported in their study that used EOG, but not the even lower value reported in the study of Berg et al. (2009) that used infrared eye-tracking system similar to ours (35, 36). It has been shown that peak velocity measures are sensitive to the sampling frequency of infrared eye-tracking systems (2). While other reasons might also play a role, the difference in the sampling frequencies between ours (1000Hz) and that of Berg et al. (2009) (240Hz) might be partly responsible for the discrepancy in the slopes (36).

### Why slower and bigger saccades and longer fixations of dogs than those of humans?

Former studies have taught us that eye movements of a species are one of the species-uniquely adapted behaviors that cannot be fully explained by their position in the phylogenetic tree (24, 25, 26). That is, similarities in other characteristics such as morphology, genes, and behavior among species do not correspond to similarities in their eye movement characteristics. Likewise, shorter phylogenetic distances among species do not guarantee more similar eye movement repertoires among them. Instead, eye movement characteristics of a species manifest how the species has adapted their eye movement behavior to the challenges in their habitat using their parallelly evolving vision-related morphology. For example, chameleons can move each eye completely independently, and this is not observed in other close reptile species, despite other similarities among them (25, 51). A similar example can be found among primate species, including humans. The study of Berg et al. (2009) and the study of Kano and Tomonaga (2011) compared eye movements of humans with those of monkeys and chimpanzees, respectively. Using natural viewing condition to draw habitual eye movement repertoires of the non-human primates, both studies reported that the saccades of the non-human primates were bigger and faster, and fixations were shorter than those of humans (36, 52). Regarding possible causes of the differences in the eye movement characteristics between non-human primates and humans, interesting speculations have been made by the two studies. Among them, Kano and Tomonaga (2011) hypothesized differences in their habitats as the selective pressure that resulted the differences in their usual eye movement behavior (52). In detail, they speculated that evolutionarily the pressure to make fast and large saccades would have been more for non-human primates than for humans, as non-human primates live in deep forest, where fast scanning of surroundings to detect other animals would greatly benefit their survival by avoiding danger and food competition (52). For the same reason, their fixations would have to be kept as short as possible to maximize the areas they can scan within a given time. Such pressure would have been less for early humans, as they lived in natural or man-made shelters and/or used animals such as dogs as their guards. Therefore, as Kano and Tomonaga (2011) pointed out, considering the widespread fast scanning strategy in primate species, it seems that the slower saccades of humans, who developed a different habitat type, have divergently evolved from that of common ancestors of all primates (52).

Then, why would dogs have the eye movement characteristics with slower and bigger saccades, and longer fixations than those of humans? What context in their environment would have worked as a selective pressure for their eye movement characteristics? Domestic dogs are direct decedents of an extinct wolf-like species, a predator, and share a great deal of behavioral and morphological traits with modern wolves (53). Yet, they uniquely have adapted to human habitat and food, where the need for such adaptation was a major selective pressure on them (54). During the domestication their original behavior repertoires in hunting, herding, and guarding would have been favored by early humans (53, 55, 56, 57, 58). Thus, it is plausible to think that the eye movement characteristics of domestic dogs would have been mainly shaped to perform visual tasks used in those human-helping activities. Major part of the visual tasks would be panoramic scanning of the horizons on the field, detection of moving objects in the periphery, and making judgments of distances to a prey, predator or possible enemy. Do their slower and bigger saccades and long fixations fit to such visual tasks? Could the visual tasks be achieved only by their eye movements? It might not be so. For example, panoramic scanning requires a wide field of view that cannot be achieved by eye movements only, even with the fastest saccades, as there is limitation in how far eyeballs can rotate. Then, how would have dogs fulfilled such visual tasks with their slower and bigger saccades and longer fixations than those of humans? Would their eye movements perhaps be assisted by other morphological characteristics? Inspired by those questions, we have looked into their vision-related morphology as possible features that would assist their eye movements. 

One of the vision-related morphology that would as- sits the eye movements of dogs is their skull shape. The axes of the two eyes of primates are parallel and their eyes are front-directed, while dog eyes are not. It is because their wolf-like skull shape makes their eyes laterally directed. The eye laterality of a species is closely related to their lifestyle, and further how they use their eyes (25). As shown in recently constructed ancient dog model, the eyes of typical dogs are directed 20 ° laterally (59, 60). With such laterality, dogs have wide field of view (240 °), which would make panoramic scanning of the horizons effortless with only a few slow and large saccades and long fixations. At the same time, their skull shape makes them to have binocular view of 30-60 °, much narrower than that of humans (140 °), and also blocked by their nose below certain height (61, 60). Further, it has been suggested that the right and left peripheral portion of their binocular view (15 ° each) would be further limited due to a lack of alpha ganglion cells in the corresponding areas of the retina (62). Therefore, their skull shape is specialized for assisting their eye movements in swift scanning and detection of moving objects on the horizons, but not for examination of small objects in close distance which requires high-quality depth perception. However, during activities such as hunting they do appear to have sufficient ability to judge distances to far away objects. They probably do so using monocular clues (60, 63).

The eye laterality of a species closely relates to how and what type of cells are distributed in their retina, and this makes the pattern of retinal cell distribution as another vision-related morphology of dogs that would assist their eye movements. In the retina, there is a high-resolution area, where retinal ganglion cells are in high density, and the aggregation of the retinal ganglion cells forms a certain shape. The shape of the area differs by species, and it relates to the lifestyle of the species (64, 65, 67). In humans and other primates, the area, called the macula (anatomical term, 5∼6 mm in diameter), is oval and has a small, round, and pit-like area, the fovea (anatomical term, ∼ 1.5 mm in diameter) in its center (68). In the fovea of the animals, the cone cells are densely packed to be efficient at scrutinizing small details within their binocular field of view. In wolves and some other animals, the area is horizontally elongated wide across the retina, and called the visual streak. The cells in the visual streak are relatively loosely distributed, except for in the *area centralis*, an area of high cone cell (cones) density that is topographically comparable to the primate fovea (62, 68, 69). Such retinal cell distribution further supports the resolution of the wide viewing field in wolves for their major foraging behavior, hunting (60). The shape of the area in average dogs resembles that of wolves, the visual streak, but the cell numbers in the area and strength of its shape is less than that of wolves. It has been speculated that the weaker form of the visual streak in dogs reflects diluted selective pressure for hunting performance put on them compared to that of wolves, as dogs adapted to human food (62).

Not only the shape of the high-resolution area in the dog retina, but also the type of photo-sensitive cells in the retina and related structures seem to assist dog eye movements as well. In primates, most cells in the central retina, in which the macula is, are cones that are crucial for color vision and high visual acuity in bright light (60, 69). The central retina of dogs contains mainly rods, the cells most sensitive to the wavelength of dim light, even in their *area centralis*, where the density of cones is the highest. Further, dog rods are also more sensitive to dim light than human rods (60). Another vision-related morphology to note is *tapetum lucidum* (shining layer of ophthalmic tissue), which lies immediately behind the retina. It is absent in many diurnal animal species like humans, and is rare in non-human primates, yet interestingly dogs, largely a diurnal species, have it (60). It makes dogs’ vision good in dim light by reflecting incoming light to increase the availability of light in the eyes, and likely also by making the wavelength of the absorbed light longer so that it gets closer to the maximum sensitivity of rods (70, 71). As a result, despite their slower eye movements, dogs’ vision outperforms primate vision in dim light which makes them better at detecting intruders that would commonly be active in the evening or dawn.

Similarly, the shape of dog pupil seems to play a role as well. Like the shapes of the skull and the high resolution area on the retina, pupil shape also has been found to be closely related to the life style of animals, especially foraging behavior and their diet pattern. Vertically elongated pupils have been correlated with being sit-and-wait predators, active day and night and horizontally elongated pupils with being preys (72). The reason behind this, outlined by Banks, Sprague, Schmoll, Parnell, & Love (2015), comes from the ophthalmic mechanisms of vertical slit pupils efficient at judging distances to the prey and horizontal pupils efficient at creating horizontally panoramic view to detect predators. Wolves and dogs, having somewhat intermediate form of foraging pattern of the two, have round pupils. In a further analyses of Banks et al. (2015), it has been found that from their last common ancestor that have subcircular pupils, wolflike canids evolved to have round pupils, differently to other canid species with vertical-slit pupils, in response to the changes in their environment and consequent foraging behavior (72).

We have described vision-related morphological characteristics of dogs that likely have co-evolved with their eye movements during their evolution. As in the experiments of Berg et al. (2009) and Kano and Tomonaga (2011), the observed eye movement characteristics of dogs in our experiment would reflect their habitual eye movement repertoires that are shaped by changes in their ecological niche. With their wide field of view that maximizes the efficiency of capturing images of their surroundings quickly, the need for them to make fast saccades with short fixations would have been minimal and less than for humans. Thus, their slower saccades and long fixations would reflect the lack of such necessity. 

As a consequence of the artificial selection by humans, dogs are morphologically and also behaviorally, the most diverse species on earth (73, 74). The artificial selective pressure focused a great deal on human-desired traits instead of their reproductive fitness as a species, making some of the traits accidentally related to their vision-related morphology. McGreevy, Grassi, & Harman (2004) reported a strong correlation of retinal ganglion cell number and distribution with skull shape, especially of nose length among different breed of dogs (75). In brachycephalic (short-nosed) dogs that have skull shape and eye positions more similar to those of primates, their retinal ganglion cells were more gathered to form a round shape, similar to that of the primate macula, instead of visual streaks that are seen in *mesocephalic* (medium-nosed) and *dolicocephalic* (long-nosed) dogs having more wolf-like skull shape. It might be that selective breeding that worked to create brachycephalic dogs also worked to make their visual performance more primate-like. Interestingly, a recent investigation of *mesocephalic* (medium-headed) dog retina reported the existence of a structure, a central bouquet of cones within the *area* *centralis*, that might work similarly to the foveola (∼ 0.2 mm in diameter) of the fovea in primates (68). As such, similar breed differences might be visible in the characteristics of the foveolar-like region as well in dogs. Variations in the existence, size and color of *tapetum lucidum* among dog breeds also has been reported (76, 77, 78). However, whether there are tiny differences in pupil shape, for example the roundness of pupils among breeds has not been investigated. Are there other accidental variations? Peichlcu (1992) reported variation in eyeball size among breeds, where larger breeds have bigger eyeballs (62). Longer axial length of eyeballs is known to relate to the visual acuity and ecological niche in animals (79). Would the accidental variations in the vision-related morphology in dogs have made variations in eye movement characteristics among dog breeds as well? Seeing the species differences in the eye movement characteristics and its relation to the differences in their morphology, it might be so. For example, differences in binocularity among breeds might somehow affect their habitual saccade speed. Testing such hypothesis was beyond the extent of our data, where almost all individual was of different breed.

### Implications on eye movement event detection algorithms

Most common velocity-based algorithms, as EyeLink 1000 algorithm require the users to set or accept predetermined threshold values for saccadic velocity and saccadic acceleration for eye movement event classification. Similarly, but differently, dispersion-based algorithms need those for minimum fixation duration and maximum dispersion. Despite their common usage, such algorithms are known to be very sensitive to the noise in the eye movement data which could make their performance poor (46). In our results, peak saccadic velocity and average saccadic velocity of dogs were significantly lower, and on the other hand, dog fixations were significantly longer than those of humans. Our results indicate that the performance of such algorithms with conventional threshold settings might be compromised when dealing with dog eye movement data, as the commonly used threshold values are based on the eye movement characteristics and data noise levels of humans. It might be worth investigating how different algorithms or threshold settings perform with dog eye movement data as similarly shown in Andersson, Larsson, Holmqvist, Stridh, & Nyström (2017).

## Limitations of the study and Future directions

Small head movements occur during most eye-tracking experiments, and the tracking mechanism of most video-based eye-tracking systems such as EyeLink1000, the use of the calculated values of P−CR, is known to cancel them out (EyeLink 1000 User Manual version 1.5.2). However, it is plausible to think that head movements bigger than the amount the system could successfully nullify could occur if the subjects are non-human animals. Possible head rotation occur during saccades would make the measured amplitudes of the saccades smaller than they actually were, and we would like to inform the readers of the possibility in our dog saccades data.

Whether different morphology across dog breeds would affect their eye movement behavior is an important topic that needs to be explored. Appropriate amount of dog individuals belonging to certain breeds would be required for such investigation. However, the amount of variation due to breed might appear in smaller scales than the variation among different species considering the largely similar environment (human households) among dogs. We hope our results provide some background for such investigation (see Table 5). While the saccade is one of the most studied eye movements, it would be interesting to explore other important eye movements in dogs. For example, microsaccades are one of the fixational eye movements which is known to indicate fixational efforts put on to a fixation on a target, and its direction indicates the direction of covert attention shift (81,82). It is less commonly observed in animals without the fovea (25, 83). Another is smooth pursuit, the eye movement that occurs when the viewer is tracking a moving target. Observation of smooth tracking with eyes only is known to be rare in non-primate animals (25).

**Table 5:** Summary table of the dog saccade and fixation statistics in the study

					Saccade											Fixation
Dog	Breed	Skull shape	sex	age	Main sequence		Carpenter's relationship		Time to peak velocity							
					Intercept (deg/s)	Slope (deg/s/deg)	Intercept (ms)	Slope (ms/deg)	Intercept (ms)	Slope (ms/deg)	Amplitude (deg)	Duration (ms)	Peak velocity (deg/s)	Velocity (deg/s)	Skewness (%)	Duration (ms)
1	Akita Inu	Brachy	f	8	99.9	14.3	61.7	3.6	23.7	1.6	9.5	96.9	236.4	12.5	41.8	2070.7
2	Australian Shepherd	Meso	m	1	78.5	18.1	59.1	3.4	23.4	1.3	8.7	90.2	225.9	12.7	37.5	1767.1
3	Border Collie	Meso	f	6	81.2	16.9	68.5	3.9	23.9	2.2	9	103.4	230.4	14.2	41.5	1144.3
4	Border Collie	Meso	f	6	71.7	13.9	66.4	4.2	22.9	2	8.8	103.2	199.1	14	38.8	2089.9
5	Border Collie	Meso	m	3	91.7	13.7	80.6	6.9	23.5	3	9	135	218.7	17.9	41.1	1890.6
6	Border Collie	Meso	f	3	95	15	79.9	4.5	23	3.5	7.6	113.3	206.9	18.4	40.2	1270.7
7	Border Collie	Meso	m	2	79.5	14	62.5	5.2	20.7	2.9	9.6	112.1	216.8	13.5	42.3	1061.2
8	Boxer	Brachy	f	3	146.8	13.2	67.9	3.2	26.2	1.6	8.7	97.5	251.4	14.7	41.6	1338.3
9	Golden Retriever	Meso	f	12	96	15.8	60.7	2.4	24.6	1.1	10.2	87	257.4	9.5	41.3	1693.1
10	Jack Russell Terrier	Meso	f	8	111.1	10.2	70	4.8	20.9	2.3	8.5	109.9	202.7	15.9	36.5	1729.1
11	mix	Meso	m	5	41.6	15	80.1	4	29.9	2.8	8.9	116	178.2	16.5	47.5	1299.1
12	mix	Meso	m	3	81	16.4	101.8	3	27.1	1.8	9.1	129.7	228.1	18.3	37.3	1481.9
13	mix	Meso	f	5	114	10.6	100.9	5.8	18	4.1	9.1	148.6	218.1	19.9	38.5	1839.5
14	mix	Meso	f	5	101.9	12	71.5	3.6	26	1.4	11	115.7	245.9	11.4	37.3	1212.2
15	Parson Russell Terrier	Meso	m	5	111.4	8.9	73.2	9.8	10.5	4.7	10	165.7	209.4	18.1	35.1	1805.4
16	Petit Brabancon	Brachy	m	7	81	17.2	62.7	4	20.9	2.5	9.2	100.7	234.4	12.6	42.4	1897.3
17	Rhodesian Ridgeback	Meso	m	6	92.5	15.6	59.4	4.3	23.3	1.7	8.2	93.4	215.9	13.9	39.3	1830.9
18	Rhodesian Ridgeback	Meso	f	5	144.3	25.2	62	4.7	18.2	2.7	8.1	98	293.5	14.5	37	1427.4
19	Siberian Husky	Meso	f	2	73.6	19.5	74.3	5.6	25.1	1.8	8	116.7	222.1	17.7	36.8	1239.7
20	Siberian Husky	Meso	f	8	77.9	14.4	66	3	25.8	1	8.7	93.6	206.9	13.3	37.1	2659.3

### Conclusion

We found similarities and differences in the eye movements, saccades and fixations between dogs and humans. We hope our findings, a piece of information about *Canidae* eye movement, help comparative analyses of eye movement across animal species and improvement of eye-tracking algorithms used for dog eye movement classification.

### Ethics and Conflict of Interest

The author(s) declare(s) that the contents of the article are in agreement with the ethics described in http://biblio.unibe.ch/portale/elibrary/BOP/jemr/ethics.html and that there is no conflict of interest regarding the publication of this paper. 

### Acknowledgements

We are grateful to all human participants and dog owners for their participation and tremendous support. We thank Sabrina Karl and Céline Delay for their hard work in training dogs, Peter Füreder, Wolfgang Berger, and Karin Bayer and Jennifer Bentlage for their excellent IT, technical, and administrative support, respectively. We thank Dr. Diederick C. Niehorster for his indispensable help during eye movement data reduction procedure. We thank the anonymous reviewers for their helpful comments. The authors would like to express their appreciation to the Cross Validated group of Stack exchange, inc. community (including the authors of R function packages) for valuable information on statistics and related computation using R. The study was funded by the Vienna Science and Technology Fund (WWTF, www.wwtf.at) No. CS11026 awarded to Dr. Zsófia Virányi and in part by No. CS11-005 awarded to Prof. Ludwig Huber.
